# Governing the research-care divide in clinical biobanking: Dutch perspectives

**DOI:** 10.1186/s40504-015-0025-z

**Published:** 2015-08-06

**Authors:** Martin Boeckhout, Conor M.W. Douglas

**Affiliations:** BBMRI-NL, Department of Human Genetics, Leiden University Medical Centre, PO Box 9600, 2300 RC Leiden, The Netherlands; Faculty of Pharmaceutical Sciences, Collaboration for Outcomes Research and Evaluation (CORE), The University of British Columbia Vancouver Campus, 4103A-2405 Wesbrook Mall, Vancouver, BC V6T 1Z3 Canada

## Abstract

Biobanking, the large-scale, systematic collection of data and tissue for open-ended research purposes, is on the rise, particularly in clinical research. The infrastructures for the systematic procurement, management and eventual use of human tissue and data are positioned between healthcare and research. However, the positioning of biobanking infrastructures and transfer of tissue and data between research and care is not an innocuous go-between. Instead, it involves changes in both domains and raises issues about how distinctions between research and care are drawn and policed. Based on an analysis of the emergence and development of clinical biobanking in the Netherlands, this article explores how processes of bio-objectification associated with biobanking arise, redefining the ways in which distinctions between research and clinical care are governed.

## Introduction

Biomedicine is frequently framed as standing at the cusp of an era of personalized medicine, an era ushered in and enabled by increasing capacities to collect and analyse huge amounts of data (Hamburg and Collins 2010). Yet achieving that transformation will first require huge infrastructural changes in biomedical research, particularly in collecting, managing and using human tissue and data in large-scale, systematic fashion (Ratto and Beaulieu 2007; Yuille et al. 2008; Park 2009; Hewitt 2011; Harris et al. 2012). Changes related to the collection of tissue and data do not solely implicate research processes, but also the provision of healthcare itself. The realization of personalized medicine is considered to require forms of biobanking that reconfigure the relationships between research and care.

In this article, we show how resources of clinical biobanking (i.e. human tissue and health data) link up and transgress commonly held distinctions between research and care in multiple ways. Recent scholarly work within science and technology studies (STS) has sought to develop a series of analytical tools for recognizing how such reconfigurations are taking place, and understanding their implications for current understandings of life (Holmberg et al. 2011; Vermeulen et al. 2012; Metzler and Webster 2011a; Hansen and Metzler 2012; Tamminen and Vermeulen 2012; Douglas et al. 2012a; Bock von Wülfingen 2012; Maeseele et al. 2013; Martinelli et al. 2013; Svalastog and Martinelli 2013; Cañada 2013). Drawing on the concepts and interpretive toolkit of bio-objectification (Vermeulen et al. 2012; Metzler and Webster 2011a; Holmberg et al. 2011), we trace how relationships and boundaries between research and clinical care are reconfigured through changes in the resources, their associated practices and the way these are governed in biomedical research. The notion of ‘bio-objectification’ calls attention to the ranges of work devoted to the exploration and fashioning of new forms of life. As these novel configurations disturb previously established boundaries, labour is undertaken to render bio-objects stable and both demarcate them and associate them with other forms and aspects of life (Holmberg et al. 2011). Such labour, involving many different practical, technical, legal and social aspects, can lead to ‘bio-objects’ such as frozen gametes that sit on the boundary of the living and non-living as they are simultaneously inanimate and sources of vitality (Tamminen 2013), or microRNA that challenges the boundary between human and non-humans as it migrates from plants to regulate mammalian genes (Chrupek et al. 2012).

Our investigation of bio-objectification processes related to clinical biobanking draws on qualitative research based in the Netherlands over a five year time period (2008–2013). Data collection consisted of semi-structured interviews with key researchers, policymakers, and others involved in establishing Dutch biobanking infrastructures and policies pertaining to them, participant observation at professional conferences, alongside analysis of public and internal documentation of a prominent large-scale national initiative in clinical biobanking – the Parelsnoer Instituut (PSI). PSI is a large initiative aimed at providing a model for collaborative clinical biobanking across clinical disciplines and medical institutions, in which University Medical Centres (UMCs) coordinate and account for the lion’s share of (relatively high-impact) biomedical research (Talmon et al. 2008; Mook 2011; Levi et al. 2013). UMCs also play a pivotal role in Dutch healthcare by providing specialized clinical care.

We use the term ‘clinical care’ loosely as a general term referring to settings and institutions of care in clinical medicine, particularly (though not exclusively) as it relates to diagnosis and health monitoring, as these are the areas most directly affected by the emergence of clinical biobanking. Our use of the bio-objectification toolkit allows for an investigation of the most salient reconfigurations involved in emergent clinical biobanking infrastructure in the Netherlands. After showing how clinical biobanking has emerged over the last decades, and how it has given rise to concerns over the relationship between research and clinical care, we turn to an analysis of associated relationships of governance. By describing how distinctions between research and clinical care are enshrined into research governance we demonstrate how bio-objectification in clinical biobanking challenges these assumptions. Finally, we investigate the implications of these challenges, and show a number of possible directions taken in policies and governance pertaining to biobanking. Specificities of the Dutch institutional landscape notwithstanding, we believe our analysis also offers broader insights into the dynamics at work at the interface of research and care in clinical biobanking. We will link up our discussion of general trends, tensions and approaches taken to academic discussions on changes in biomedical research governance more broadly.

## Tissue and data for research and its relationships to care

The emergence of clinical biobanking is associated with general shifts in biomedical research towards an investigation of the molecular level to understand and intervene in mechanisms of disease, particularly with the uptake of genomics in in clinical research and medicine. In turn, those shifts bring with them a hugely different role for human tissue and data as well as major changes in the ways in which tissue and data moves between research and care. These shifts provide a novel occasion for investigating relationships between research and care. In medical sociology and STS the relationships between research and care have been explored in a number of ways, particularly through addressing the ways in which medical uncertainties are dealt with by practitioners and researchers (Fox 1997; Timmermans and Angell 2001; Alderson 2014); the consequences of intertwining research and care at the level of clinical practice (Löwy 1996; Timmermans 2010; Wadmann and Hoeyer 2014); the role of clinical trials as a constitutive component of clinical cancer care (Keating and Cambrosio 2012); as well as the ways in which changing practices and processes of research and drug development affect the organization and practice of clinical care and public health (Fisher 2009; Petryna 2009). Our research touches on the latter focus in particular. Analogously to the ‘experimentalization’ of clinical care for drug development, the reconfiguration of clinical care to accommodate biobanking can be understood as a way in which care practices are changed in order to feed into and accommodate broader research objectives.

Healthcare traditionally serves as the prime resource for biomedical research as a setting for recruiting patients as research subjects as well as a source of tissue and data. The interweaving of research and care also played a role in the emergence of modern medicine, as Michel Foucault argues in his classic study on the emergence of the modern clinic in which patients suffering from similar symptoms were assembled in a way that enabled them to be submitted more systematically to a ‘clinical gaze’ (Foucault 2012). Foucault shows how distinct constitutions of the patient emerge with new ways of thinking about medicine and sickness as well as new technologies and techniques for investigating and recording the body. The emergence of new tools and techniques for examining particular organs went hand-in-glove with an associated disciplinary compartmentalization of the body, as well as an institutional sequestration of bodies in the clinic. In this respect, collection of -and research on- human tissue and data in relation to clinical medicine is far from novel as such. For instance, there is a long history of changing techniques and forms of research building on isolating, banking and manipulating human tissue for research purposes (Landecker 2007), and residual use of human tissue and data procured for medical purposes is common in modern medicine as well. Such uses involve medical files, but also blood leftover from diagnostic tests or excised tumor tissue. These are facilitated by infrastructures such as tissue archives in pathology that are set up for healthcare purposes, disease-related patient registries, and archives of dried blood spot cards collected through newborn screening for congenital defects.

The role played by human tissue and data, and the value attached to it, in research is now shifting along with novel approaches and techniques of biomedicine. Instead of focusing on causal mechanisms, health and disease are frequently understood nowadays in terms of risks and aiming for differentiation and stratification of diseases and disease populations. In order to accommodate that shift, resource provision for research has changed dramatically in scale, scope and systematic nature over the last decades. In seeking to exploit the potential of genomics and other molecular analytical techniques, increasing emphases on differentiation and stratification of target objectives and populations have emerged, while challenges in establishing statistically significant associations between diseases and disease markers require data of ever larger target populations – both healthy subjects as well as patients (Burton et al. 2009). While often grouped together under the heading of ‘personalized medicine’, current approaches to biomedical research therefore involve more than just individualized, stratified and differentiated forms of intervention, but also new forms of population-level surveillance (Raman and Tutton 2010). The emergence of biobanking is considered a chief enabling factor for these shifts.

Population-based biobanking set up specifically for research purposes have received considerable attention in studies devoted to ethical, legal and social aspects of biobanking (Häyry et al. 2007; Gottweis and Petersen 2008; Dierickx and Borry 2009; Solbakk et al. 2009; Kaye and Stranger 2012). However, equally large changes in relation to biobanking are afoot in the practices and institutional settings of healthcare. The advent of molecular medicine both builds on and transforms existing ways in which body parts and data derived from them are procured, stored and used. Systematic, so-called, ’repurposing strategies’ are now considered for most retrospective collections of tissue and data collected for purposes of healthcare (cf. Mitchell 2012). Prominent examples in the Netherlands include proposals for systematic use of dried blood spot cards for research, efforts aimed at increasing research opportunities from pathology archives and infrastructure, as well as initiatives in clinical biobanking (Casparie et al. 2007; Talmon et al. 2008; Dutch Forum for Biotechnology and Genetics 2010; National Institute for Public Health and the Environment (RIVM). 2010; Douglas et al. 2012a; Douglas et al. 2012b). The Dutch branch of the European biobanking platform BBMRI has been providing funding to projects aimed at systematizing and upgrading existing collections for genomics research since 2009 (Brandsma et al. 2012). The project is now in its second phase, which will run until at least 2017.

Therefore, while human tissue, data, and the bodies of patients these are derived from have traditionally served as boundary objects between research and care, those linkages are now formalized, systemized, and institutionalized on a much larger scale into basic routines of clinical care and molecular medicine. This is particularly true of healthcare taking place in academic centres or university teaching hospitals. PSI is a particularly prominent national initiative in the Netherlands in this respect, linking all eight UMCs with the goal of standardizing the procurement, management and distribution of samples from patients in academic hospitals for a number of different areas of disease. At present, over thirteen clinical specialties have joined up in this model to collaborate in the coordinated provision of human tissue and data for research purposes. Through PSI, these medical centres are taking up the task of professionalizing and systematizing the ways in which tissue and data are managed locally for subsequent research. This has also stimulated the establishment of new institution-wide biobanking facilities that dovetail with existing pathology and clinical chemistry facilities (cf. for instance the Radboud biobank in Manders et al. 2014).

These Dutch initiatives are by no means unique in the world. In Denmark, for instance, opportunities for exploiting leftover dried blood spot cards from neonatal screening for genomics research are being considered (Sørensen et al. 2007). Other initiatives for coordinating provision and access (particularly tumor) samples in the United States and across Europe as well (Riegman et al. 2006; Mitchell 2012; Gottesman et al. 2013; Reichel et al. 2014). Prospective initiatives with comparable aims are emerging at academic healthcare institutions across the globe as well as at the field level around specific diseases (European Commission 2012; Mora et al. 2014).

Below we detail how the emergence of clinical biobanking is reconfiguring relationships and interactions within and between research and care. By examining the procurement of research material in care settings, the alteration in clinical practice due to research protocols, and the routinization of patient participation in research through the analytical lens of bio-objectification, we show how these reconfigurations are currently taking place. With that description in place we will then move to a discussion of the socio-political and governance implications of those shifts.

## Processes of bio-objectification in clinical biobanking

The emergence of clinical biobanking has gone hand in hand with a blurring of the boundaries between clinical care and medical research. Specific components of that blurring can be understood as bio-objectification, a process through which novel personal and biological entities (in our case tissue and data) come into being and result in a reframing of the roles, responsibilities and agency of other parties, entities and institutions involved (L. Eriksson and Webster 2015). In particular, we see three forms of bio-objectification taking place in clinical biobanking, each of which is challenging conventional boundaries between biomedical research and clinical care.

First of all, data and tissue initially procured for and circulating in contexts of academic clinical care is now often framed and systematically formatted as also catering to potential research purposes. For instance, according to one of the chief instigators of PSI, Daniel Hommes, the integration of care and research on the level of data is a chief imperative for clinical researchers working in academia (Hommes 2007). Hommes’ vision subsequently became a driving force in the establishment of PSI as well as related local initiatives in clinical biobanking. For clinical biobanking, facilitating such integration involves huge amounts of work aimed at standardization and harmonization of data and tissue provision as well as efforts aimed at establishing quality control, certification of workflows, substantial and procedural benchmarks for data and tissue collection and management and evidence-based data models (Riegman et al. 2006; Mook 2011)). Slightly different attempts at integrating healthcare data for research are made in projects aiming at large-scale systematic integration of medical data infrastructure into biomedical research, such as the controversial UK care.data project (Carter et al. 2015).

In order to achieve such close integration and harmonization, a second, related process of bio-objectification is also involved. The integration of care and research at the level of data and tissue does not just involve changes in the ways in which data and tissue are collected for research; rather, it also implies changes in the uses of tissue and data for purposes of care. For instance, in the context of PSI, clinician-researchers established so-called minimal datasets which specify how and what kinds of data would be collected of what patients. These were subsequently institutionalized into all clinical routines across participating UMCs. Settling on minimal datasets for purposes of research also involved settling details of how data would be collected in the context of care. Clinician-researchers across different institutions had to settle on questions such as whether blood samples would be collected from sober patients only. While many such changes may seem mundane (even if complex to change in a coordinated fashion), other changes also involved the establishment of novel and state-of-the-art invasive routines across multiple care settings. For instance, PSI catalyzed the introduction of routine collection of cerebro-spinal fluid for the purposes of Alzheimer diagnostics in UMCs (Douglas and Scheltens 2014). In cases such as this one, research processes are impacting the provision of clinical care, through novel standardized routines for the collection and storage of biomaterial and data on a nation-wide scale.

A third process of bio-objectification relates to the patients participating in these clinical biobanking endeavors and the roles they are expected to take up vis-à-vis the tissue and data procured from them. Through large-scale forms of resource provision embedded into practical routines and infrastructures for healthcare, patients are turned into regular contributors to the clinical research enterprise. This is reflected in terminology involved to describe their role. Instead of the use of language such as ‘research subjects’, contributing human tissue and data is now often framed as an act of ‘donating’, which a term previously reserved for more tangible donations dedicated to others‘ well-being such as through blood donations (Tutton 2002). A case in point is that in 2011 Dutch professional guidelines for responsible use of human tissue in biomedical research routinely speak of ‘donors’ and ‘donations’; yet, in 2001 the terminology used was ‘*betrokkene*’ (i.e. someone who’s involved) (Federatie van Medisch-Wetenschappelijke Verenigingen (FEDERA) 2001; Federatie van Medisch-Wetenschappelijke Verenigingen (Federa) (2011)). Some scholars have referred to this process as involving new forms of ‘clinical’ and ‘immaterial labor’. At the same time, the labour performed by most donors is also minimized and made invisible by integrating it into routine aspects of care (Mitchell and Waldby 2010; Mitchell 2012). In order to achieve high rates of donation, the success of clinical biobanking is considered to depend on its unobtrusiveness and on not being seen to overburden patients in their donations. This is reflected in concerted efforts in PSI to minimize the work and time expended on biobanking for patients, research nurses and clinicians by integrating tissue and data procurement as efficiently as possible in day-to-day clinical care. These adjustments in clinical routines, which also involve mundane aspects such as training of research nurses and timing of clinical appointments, are forms of bio-objectification that allow for patients’ data and tissue to be swiftly transformed into “workable epistemic objects” (Eriksson and Webster 2015).

## How clinical biobanking challenges research governance

Through these processes, clinical biobanking poses challenges to clinical research governance. In a number of ways, such governance assumes and is aimed at enacting and enforcing distinctions and boundaries between research and care. As we have noted elsewhere,

Establishing and maintaining firm boundaries in biomedical practices are crucially important activities for establishing legal rights and responsibilities, as well as the navigation of routes to regulatory approval of new medicines and products. Classifications delineate what is and is not acceptable within biomedicine, which has knock-on effects in terms of how science, health care, and biomedical research will be structured, organized and funded. However, when such boundaries are breached and classifications begin to breakdown, questions are raised about how biomedicine will be governed. (Douglas et al. 2012a)

The first issue raised by the bio-objectification of clinical biobanking relates to the core principle underpinning the ethics of human subjects research: the protection of the autonomy of research participants. Distinguishing sharply between research participation and receiving care is widely considered part and parcel of such protection. The Dutch Law on Research Involving Human Subjects, for one, imposes a regulatory check on medical research on a project-by-project basis according to three basic criteria:

- Human subjects research needs to be aimed at a specific, circumscribed goal, laid down in a protocol;
- Each research subject needs to be free to decide about participation informed about and consent to potential risks and benefits in advance of their participation, through providing informed consent;
- Research projects require ethics review, involving approval of protocol and consent procedure of an ethics review board (ERB) before the start of research as well as more marginal monitoring of possible safety breaches throughout the project.

Each of these criteria presuppose and serve to reinforce distinctions between research and care, which are destabilized by the objects and collection routines emergent in clinical biobanking. For instance, research protocols are directed at delimiting the scope of research in both substance and time, while explicating and justifying potential risks associated with that research to participants. Informed consent is a way of framing participation in research as an issue of individual choice made on a well-informed basis related to circumscribed research objectives. Finally, ethics approval of both aspects serves as a check of the specific risks and research potential of each research objective taken on its own. The institutionalization of clinical biobanking into practices and infrastructures of healthcare challenges the project-based modes of research ethics regulation, and hence represent a significant governance challenge. The open-ended nature of biobanking is considered a crucial concern in this respect, and is a point that is raised time and again in discussions over the nature of informed consent, (e.g. J. Kaye et al. 2011; Hoeyer 2008; Spencer et al. 2012; Hallinan and Friedewald 2015).

This issue has also provided a powerful catalyst for the development of models of governance that would look to endure over longer periods of time (Knoppers 2009). However, such models of governance are complicated by the extent to which clinical biobanking initiatives are organized as complex nested arrangements, often involving overlapping organizational responsibilities for various aspects of tissue and data processing. This is a second challenge mounted by processes of bio-objectification. PSI, for one, draws together multiple departments located in different institutions collaborating on a number of specific disease areas. Clinical specialties from different academic hospitals collaborate in disease-specific entities called ‘Pearls’, while each academic hospital separately provides institution-specific logistical and technical facilities to its participating departments. The need for coordination between disease areas, medical institutions, as well as individual departments leaves considerable leeway for variation and conflict on many aspects of the initiative as a whole with respect to aspects such as the formats in which data is collected, issuance of data and tissue requests from the biobank, ethical and legal affairs, quality control, communications, finance, information communication technology and information security. The governance relationships between all these organizational entities are complex, diverse and subject to ongoing negotiation and modification. Such complex, nested organizational arrangements complicate project-based model of research ethics regulation, since ethics review for clinical research traditionally stresses the need to review the proportionality of research potential and risks upfront. In the case of clinical biobanking, ERBs find such a check on proportionality complicated by the time span passing between procurement and use of data and tissue for specific research projects. This became an issue when PSI sought ethics approval. ERBs and policymakers considered there to be a lack of legal basis for ethics review for projects lacking specific research objectives. Moreover, the fact that local standards of care provide an informal benchmark for both researchers and ERB members against which to compare the invasiveness of research interventions also complicates checks on proportionality. Several components of PSI involved not just the procurement of additional tissue and data for research, but also extensive changes in local standards and procedures of care, complicating the allocation of the burden of procedures to either research (in which case proportionality is an issue for ERBs) or care (in which case it technically is not). The previously mentioned example of cerebro-spinal fluid (CSF) provides a case in point. Procurement of such fluid, which had been incorporated into one leading institution’s diagnostic routines for neurodegenerative diseases for a while already, was adopted by other clinician-researchers in the course of participating in PSI (Douglas and Scheltens 2014). After protracted discussions, local ERBs eventually settled on compromises which allowed the initiative to continue, but with additional liability insurance for human subjects research in place in a number of the locations where diagnosis using CSF had not previously been included in clinical routines.

Thirdly, practices of residual use of human tissue and data procured in the context of healthcare often do not directly fall under the remit of most clinical research legislation. Historically, human tissue and data were often regarded as a kind of waste which could be regarded as an impersonal good (Tupasela 2011). Even where personal rights in such resources were involved, current privacy legislation often contains provisos for so-called research exemptions. In this way, a distinction between research and care is upheld by depersonalizing the use of residual tissue and data in research and processing such resources only in aggregate form. As discussed above, such ways of drawing boundaries between research and care no longer apply in clinical biobanking. The boundaries are blurred by design, undercutting any sharp division between data for research and data for care. One area in which this blurring plays up clearly is in current debates over how to deal with the feedback of incidental findings. Many ethicists and legal scholars have argued that researchers and biobanks have duties and responsibilities towards participants and donors with regards to incidental findings generated from banked tissue and data. For instance, Wolf and others consider that “findings that are analytically valid, reveal an established and substantial risk of a serious health condition, and are clinically actionable should generally be offered to consenting contributors”(Wolf et al. 2012). However, it is often unclear on whom this responsibility specifically falls, and this may require altering conventional roles and duties of researchers. This could extend researchers’ medical responsibilities and would consequently also raise further governance challenges concerning the delineation of their role and remit in research and care. Even the question whether findings should still be considered ‘incidental’ given the systematic exploration of data and tissue will come up for debate. Irrespective of if most genomic variants may currently by-and-large seem of unclear significance, such findings are likely to be commonplace in some clinical settings (i.e. genetic diagnostics) and will eventually become more commonplace as similar analytical techniques are adopted in other clinical areas as well. Moreover, once personal tissue and data collected in care settings are processed for open-ended purposes over indeterminate time frames, research data may become a source of data with potential clinical significance as well. Once healthcare practices are modified to accommodate the provision of clinical data for research purposes, qualitative distinctions between clinical and research data are less likely to form a barrier to such feedback.

Fourthly, challenges related to the blurred boundary between research and care in clinical biobanking also emerge with respect to the rights of participants and the vexed issue of informed consent. An avalanche of academic literature on informed consent in biobanking has appeared over the last decade (Clayton 2005; S. Eriksson and Helgesson 2005; Salvaterra et al. 2008; Hofmann 2009; Allen and Mcnamara 2011; Spencer et al. 2012). Research legislation is often considered an impediment to, or safeguard against (as some ethicists would hold), ‘broad’ and generic forms of informed consent. This challenge is further compounded by the fact that consent is designed to regulate the rights of research participants and the obligations of researchers vis-a-vis them. Clinical biobanking often involves fairly diffuse relationships, relating to responsibilities to safeguard privacy over time as well as responsibilities relating to the integration of research into care. Consent serves a different role in such a constellation and it becomes a placeholder for a much more diffuse set of entitlements and expectations regarding the control individuals should hold over their data and tissue within clinical biobanking infrastructures. In the Netherlands these issues were raised during ethics review of PSI. ERBs delimited the scope of consent, particularly by requiring subsequent ethics approval of projects applying for the use of tissue and data from PSI. At the same time, patients’ role in such consent procedures remained restricted to a generic approval at the point of collection of tissue and data (Boeckhout et al. 2010).

## Challenges to governance: reinstituting or flexibly managing distinctions between research and care?

Clinical biobanks, and the tissue and data brought into circulation through them, uncomfortably straddle governance regimes of clinical medicine and biomedical research and are facing renegotiations of the terms under which biological material and data are collected. Divergent approaches to dealing with these challenges of bio-objectification can be discerned. While some approaches aim at purification and the re-establishment of boundaries between research and care through updates and extensions of existing modes of governance, others aim at hybridization, flexibly managing the traffic across the divide. In practice, both are coined next to one another, providing an additional source of conflict.

One particularly salient challenge in this respect relates to the individual feedback of findings. At stake in such discussions are a series of ethical, legal, economic and medical questions about what kinds of outcomes of research should be reported back to individual contributors of data and tissue and under what circumstances. Considering the diversity of kinds of data and tissue, contexts of procurement and kinds of research involved, this makes for a fraught discussion (Hoeyer 2010; Wolf et al. 2012; Wolf 2013; Thorogood et al. 2014). The issue is complicated further by the fact that similar debates on the issue of reporting back results from techniques such as imaging and whole-genome sequencing in clinical and diagnostic settings remain unresolved in the Netherlands as elsewhere (Health Council of The Netherlands 2014; Health Council of The Netherlands 2015). Various proposals to establish protocols and guidelines to deal with the issue have been made. A P3G consensus document proposed that every biobank should at least have established some policy on how incidental findings would be handled, but the content of such policies remains very much a matter of dispute (Cornel 2013; Viberg et al. 2014). Although some biobanks have developed preliminary policies, the majority of biobanks in the Netherlands have not done so up to now (E. Vermeulen et al. 2014). Some lawyers and ethicists argue forcefully for policies limited to only the most clear-cut, acute ‘clinically actionable’ cases, minimizing the medical responsibilities involved (Clayton and McGuire 2012). Dutch researchers have argued publicly and in academic debate for substantive restrictions on the clinical relevance of data. Genetic epidemiologist Cecile Janssens pointed to the limited quality control for research data and interpretation of genomics data (Janssens 2014). Community geneticists involved in the European Society for Human Genetics (ESHG) have suggested that researchers employ data filters designed to screen off potentially significant clinical findings for particular research investigations (van El et al. 2013). Others, such as medical ethicist Annelien Bredenoord, advocate and experiment with more hybrid policies for dealing with and reporting back different ranges of findings to those who are interested, including findings of only potential personal significance such as slight changes in genetic risk susceptibility or findings which might inform reproductive decisions (Bredenoord et al. 2011). Dutch population-based biobanking initiatives such as LifeLines and the Netherlands Twin Registry are also experimenting with reporting back preliminary screening results and survey findings over time as a means to engage with their participants. Responding to a keynote lecture of 23AndMe’s then senior medical director at a major biobanking conference (Hands On Biobanks) in November 2013, multiple researchers considered 23AndMe’s policy on data sharing an example to be followed. The severe ethical and legal conundrums surrounding 23AndMe’s ways of feeding back findings notwithstanding, many considered their model to be attractive, not least because of the kind of involvement and interest on the part of ‘citizen scientists’ such feedback of data may invoke (Prainsack 2011; Wyatt et al. 2013).

‘Purification’ measures aimed at disentangling effects and attachments of tissue and data in care and research are also taken to adapt existing modes of ethics review to the regulation of organizational forms of clinical biobanking. Uncertainties regarding the legal status of biobanking vis-a-vis medical research legislation notwithstanding, Dutch ERBs have gone ahead in reviewing proposals for biobanking on a project-by-project basis similar to ethics review of clinical trials. Clinical biobanking initiatives are now required by ERBs to explicate their research methods and objectives in a more or less circumscribed way in a protocol, with informed consent specific to the terms laid down in such a protocol. ERB monitoring of such projects then extends to subsequent use through ethics review of projects drawing on collected tissue and data. This way of holding biobanking initiatives to account by circumscribing research objectives and monitoring of progress brings clinical biobanking back into the fold of research ethics (Boeckhout et al. 2010). In practice, however, such regulatory strategies leave considerable leeway in the manner in which clinical biobanking initiatives are governed. Within PSI many aspects of governance, such as those relating to access policies and substantive choices with respect to the kinds of data to be collected, are dealt with through consultation and management at the organizational level, with ERBs playing a minor oversight role. Such hybrid forms of self-governance imply more flexible forms of governance of the boundaries between research and care.

Similarly, both purification and hybridization approaches are at work in relation to the donors’ rights and entitlements to tissue and data. Clinical biobanking initiatives require informed consent of their participants of varying scope and specificity. Blanket consent is generally not accepted by ERBs. Instead, ERBs require the scope of consent to be circumscribed to a particular research area, while remaining linked to ongoing oversight in actual research uses. Within such a framework of ethics approval, consent still by and large serves the same role that it does in clinical research more generally (i.e. as a means to state upfront what research is about and as a device to circumscribe and delimit subsequent entitlements and expectations of patients in contributing to a research endeavor). Subsequent control of human tissue and data in such a model of consent is usually limited to a right to withdraw data and tissue for further use. More recently, however, proposals for so-called ‘dynamic consent’ have also been made that take a more hybrid approach (J. Kaye et al. 2011; J. Kaye et al. 2015). According to its proponents, dynamic consent may serve a role in programmes to make research more ‘patient-centric’, enabling patients to engage more actively in the research process themselves as well as granting them more authority over their tissue and data over time. Arguments for such active models of patient participation in research often dovetail with arguments for participatory healthcare supported by contemporary information communication technology-driven healthcare capable of facilitating both at the same time (Stein and Terry 2013). At the same time, however, fierce protests in Europe from the medical research camps over proposed new data privacy legislation have emerged (Fears et al. 2014; Hallinan and Friedewald 2015). The protests are aimed particularly against explicit, detailed consent requirements for secondary use of medical data for medical research. According to medical researchers, limiting the scope of research exemptions in data protection legislation would severely hamper biomedical research. Such exemptions thereby enact a principally different way of policing the traffic between biomedical research and healthcare. Instead of blurring the boundary on the level of individual donors, these arguments consider research as a public good relying on care for resources: ‘In many studies that will be affected [by new data privacy legislation], individuals have voluntarily given broad consent for their data to be used in research to further our understanding of society, health and disease’ (Academy of Medical Sciences et al. 2014). The chief value put in play to justify such broad forms of consent, oriented as these are towards enhancing purported collective benefits of research, is not autonomy but a form of solidarity with patients mediated through biobanking research – solidarity which serves to shield biomedical research from overly grand responsibilities vis-à-vis individual donors.

## Conclusion: clinical biobanking, bio-objectification and the governance of the research-care boundary

Generally, changing relationships between research and care have received relatively little explicit consideration in academic reflection on biobank governance. One of the central values of the bio-objectification toolkit is that it helps us make visible in-between forms of life, or entities such as tissue and data – or clinical biobanking infrastructure more generally – that straddle conventional conceptual distinctions and practical and institutional boundaries. Our analysis shows that a number of bio-objectification processes are ongoing and actively pursued, resulting in new ‘epistemic objects’ which mediate between practices of research and clinical care The formatting of data and tissue collected in contexts of care in order to cater to potential research purposes; the entanglement of new forms of resource provision in existing processes and practices of clinical care; and the routinization of turning patients into contributors to the clinical research enterprise: each of these processes present important issues of governance pertaining to the relationships between research and care. As we have outlined here, these challenges emanate from the fact that dominant modes of clinical research governance assume research and care as morally and practically distinct sets of activities, and attempt to constitute them as such. As a consequence, diverse approaches have been deployed in an attempt to address these challenges and “establish a stabilized field of inquiry that addresses regulatory and wider challenges” (L. Eriksson and Webster 2015).

We have shown here how clinical biobanking is accompanied by changes in the constitution of healthcare leading to novel, systematic infrastructural couplings between research and care mediated by human tissue and data. Clinical biobanking infrastructures underpinning data-driven research are accompanied with medical responsibilities for those involved in using and managing the data and tissue circulating therein. The tensions raised are dealt with in different, at times conflicting ways: by re-establishing and re-purifying distinctions between research and care within the novel setting of clinical biobanking, but also by actively embracing the hybrid nature of clinical biobanking between research and care by flexibly managing the intermingling of both domains. Given the multiple ways in which the governance issues related to the bio-objectification of clinical biobanks can be and are in practice addressed, governance for clinical biobanking is likely to remain a dynamic and heterogeneous field. While different approaches to processes of bio-objectification may be compatible at times, they depart from opposing philosophies. Underlying these different responses are questions and visions of how healthcare and research should be related, as well as questions about how the contribution research makes to healthcare should be understood. Is it a common good contributing to the well-being of anonymous others over the longer term, or as a good closely linked to the fate of patient-participants? In consequence, moving the debates outlined here forward also requires asking political and social questions about what goals biobanking and biobank governance should serve, and the kinds of accountability required to foster them. From various angles, scholars have proposed alternative understandings and principles underpinning biobank governance based more overtly on concepts of solidarity and the public good (Knoppers and Chadwick 2005; Prainsack and Buyx 2013).

In this sense, clinical biobanking is but one example of broader challenges in contemporary biomedicine. The ongoing transformation of academic clinical care through -and for- biobanking research represents another way in which biomedicine is increasingly turned into an ‘experimental field’ (Petryna 2009). Instead of clinical trials moving to countries in which access to healthcare is a relatively scarce commodity, however, it is medicine in affluent societies which may be transformed primarily for data- and tissue-intensive clinical research. This is a further way in which relationships between science and society become ever more complex and intermingled, a process accompanied by uncertainties, conflicts, new political fault lines and challenges, but also by new forms of governance. We believe that issues of governance pertaining to the relationships between research and care in clinical biobanking and beyond should be explored further in that direction.

## Abbreviations

ERB: Ethics review board
PSI: Parelsnoer institute
UMC: University medical centre
